# Supplementary material for: SARS-CoV-2 Infection in Venezuelan Pediatric Patients—A Single Center Prospective Observational Study
Source: Biomedicines. 2023 May 9;11(5):1409. doi: 10.3390/biomedicines11051409 (PMC10216668; doi:10.3390/biomedicines11051409)
Supplement: Supplementary file 1 [file biomedicines-11-01409-s001.zip › biomedicines-2383348-supplementary.pdf]

| *Pacient* | *clinical severity* | *Nutrition Diagnosis* | *comorbidity* | *complications* |
|---|---|---|---|---|
| 1 | severe | NORMAL | NO | bacterial coinfection |
| 2 | severe | Malnutrition | asthma | MIS- C |
| 3 | moderate | NORMAL | NO | NO |
| 4 | mild | NORMAL | NO | NO |
| 5 | moderate | Malnutrition | NO | NO |
| 6 | moderate | NORMAL | NO | MIS- C |
| 7 | mild | NORMAL | NO | NO |
| 8 | severe | Malnutrition | NO | bacterial coinfection |
| 9 | moderate | NORMAL | asthma | MIS- C |
| 10 | moderate | NORMAL | asthma | MIS- C |
| 11 | moderate | Overweight-obese | NO | NO |
| 12 | moderate | NORMAL | NO | bacterial coinfection |
| 13 | severe | NORMAL | nephropathy | MIS- C |
| 14 | moderate | NORMAL | NO | bacterial coinfection |
| 15 | moderate | NORMAL | NO | NO |
| 16 | mild | Malnutrition | NO | NO |
| 17 | mild | NORMAL | NO | MIS- C |
| 18 | mild | NORMAL | NO | viral coinfection |
| 19 | severe | Malnutrition | CANCER | NO |
| 20 | mild | NORMAL | NO | MIS- C |
| 21 | severe | Malnutrition | NO | MIS- C |
| 22 | severe | NORMAL | NO | MIS- C |
| 23 | severe | Malnutrition | nephropathy | NO |
| 24 | moderate | Malnutrition | NO | NO |
| 25 | moderate | NORMAL | NO | NO |
| 26 | mild | NORMAL | NO | MIS- C |
| 27 | moderate | Malnutrition | NO | NO |
| 28 | mild | Malnutrition | CANCER | NO |
| 29 | moderate | Malnutrition | NO | NO |
| 30 | moderate | Malnutrition | asthma | NO |
| 31 | mild | NORMAL | NO | NO |
| 32 | moderate | Overweight-obese | NO | NO |
| 33 | mild | Overweight-obese | NO | MIS- C |
| 34 | mild | NORMAL | NO | MIS- C |
| 35 | mild | Malnutrition | NO | NO |
| 36 | mild | NORMAL | nephropathy | MIS- C |
| 37 | mild | Malnutrition | CANCER | MIS- C |
| 38 | mild | NORMAL | NO | NO |
| 39 | moderate | NORMAL | NO | MIS- C |
| 40 | moderate | NORMAL | emoglobinopath | MIS- C |
| 41 | moderate | NORMAL | NO | bacterial coinfection |
| 42 | mild | NORMAL | NO | NO |
| 43 | mild | NORMAL | NO | NO |
| 44 | mild | Malnutrition | e I diabetes mell | NO |

| | | | | |
|---|---|---|---|---|
| 45 | mild | NORMAL | asthma | NO |
| 46 | mild | Malnutrition | NO | NO |
| 47 | moderate | Malnutrition | NO | NO |
| 48 | mild | NORMAL | NO | NO |
| 49 | mild | Malnutrition | nephropathy | NO |
| 50 | mild | NORMAL | NO | NO |
| 51 | mild | NORMAL | NO | MIS- C |
| 52 | mild | NORMAL | NO | NO |
| 53 | mild | NORMAL | NO | NO |
| 54 | mild | NORMAL | NO | NO |
| 55 | mild | NORMAL | emoglobinopath | NO |
| 56 | mild | NORMAL | NO | NO |
| 57 | mild | NORMAL | asthma | NO |
| 58 | mild | Malnutrition | NO | MIS- C |
| 59 | moderate | NORMAL | NO | MIS- C |
| 60 | mild | NORMAL | NO | NO |
| 61 | moderate | Malnutrition | NO | MIS- C |
| 62 | moderate | Malnutrition | NO | MIS- C |
| 63 | moderate | NORMAL | NO | NO |
| 64 | mild | Malnutrition | CANCER | NO |
| 65 | moderate | Malnutrition | asthma | bacterial coinfection |
| 66 | mild | NORMAL | NO | NO |
| 67 | mild | Malnutrition | NO | NO |
| 68 | mild | NORMAL | NO | NO |
| 69 | moderate | NORMAL | NO | NO |
| 70 | mild | Malnutrition | CANCER | NO |
| 71 | mild | NORMAL | nephropathy | NO |
| 72 | moderate | NORMAL | NO | MIS- C |

| ***treatment*** | ***sex*** | ***age group*** |
|---|---|---|
| dexamethasone | male | Infant |
| HYDROCORTISONE | male | Preschooler |
| dexamethasone | female | Preschooler |
| no | female | Infant |
| HYDROCORTISONE | female | Infant |
| HYDROCORTISONE | female | Infant |
| dexamethasone | female | Infant |
| dexamethasone | male | Infant |
| dexamethasone | female | School-age |
| dexamethasone | female | Preschooler |
| dexamethasone | female | Adolescent |
| no | female | Infant |
| dexamethasone | male | Preschooler |
| no | female | School-age |
| dexamethasone | male | Infant |
| no | male | Preschooler |
| dexamethasone | female | Infant |
| no | female | Preschooler |
| dexamethasone | male | Preschooler |
| dexamethasone | female | Infant |
| METHYLPREDNISOLONE | female | Preschooler |
| dexamethasone | male | Infant |
| dexamethasone | male | Adolescent |
| METHYLPREDNISOLONE | female | Preschooler |
| dexamethasone | female | Infant |
| dexamethasone | male | Infant |
| METHYLPREDNISOLONE | male | Infant |
| no | male | School-age |
| METHYLPREDNISOLONE | female | Infant |
| dexamethasone | female | School-age |
| no | female | Preschooler |
| dexamethasone | male | Infant |
| dexamethasone | male | Adolescent |
| dexamethasone | male | Infant |
| no | female | School-age |
| dexamethasone | male | Preschooler |
| Dexamethasone +immunog | female | School-age |
| no | female | Infant |
| Dexamethasone +immunog | male | Preschooler |
| dexamethasone | male | Preschooler |
| dexamethasone | male | Infant |
| no | female | School-age |
| no | male | Adolescent |
| no | female | School-age |

| | | |
|---|---|---|
| no | male | Adolescent |
| no | male | School-age |
| dexamethasone | female | Preschooler |
| no | male | School-age |
| no | male | Adolescent |
| no | female | School-age |
| dexamethasone | female | Preschooler |
| no | female | Infant |
| no | female | Preschooler |
| no | female | School-age |
| no | female | School-age |
| no | male | Infant |
| no | male | Adolescent |
| Dexamethasone +immunog | male | School-age |
| HYDROCORTISONE | female | Infant |
| no | male | Infant |
| dexamethasone | female | Preschooler |
| HYDROCORTISONE | male | Preschooler |
| dexamethasone | male | Infant |
| NO | male | School-age |
| dexamethasone | female | School-age |
| no | male | Preschooler |
| no | male | School-age |
| no | female | Preschooler |
| dexamethasone | male | Infant |
| no | female | Preschooler |
| no | female | Adolescent |
| dexamethasone | female | Infant |

| *symptoms* |
|---|
| fever, cough and respiratory distress |
| fever, cough and respiratory distress |
| fever and diarrhea |
| fever and cough |
| fever, cough and diarrhea |
| fever, cough and vomiting |
| fever and cough |
| fever, cough and respiratory distress |
| fever, cough and respiratory distress |
| fever, cough and respiratory distress |
| fever, cough and respiratory distress |
| fever, cough and respiratory distress |
| fever, cough and respiratory distress |
| fever, cough and respiratory distress |
| fever, cough and respiratory distress |
| fever and cough |
| cough |
| fever and diarrhea |
| fever, cough and respiratory distress |
| fever |
| fever, cough and respiratory distress |
| fever, cough and diarrhea |
| fever, cough and respiratory distress |
| fever, cough and respiratory distress |
| fever, cough and diarrhea |
| fever |
| fever, cough and diarrhea |
| fever and cough |
| fever, cough and respiratory distress |
| fever, cough and respiratory distress |
| fever and cough |
| fever, cough and diarrhea |
| fever and cough |
| fever and cough |
| fever and cough |
| fever and diarrhea |
| fever and cough |
| fever and cough |
| fever and diarrhea |
| fever, cough and diarrhea |
| fever, cough and respiratory distress |
| fever, cough and respiratory distress |
| fever |
| fever and cough |

| |
|---|
| fever and cough |
| fever |
| fever and diarrhea |
| fever and cough |
| fever, cough and diarrhea |
| fever |
| fever, cough and diarrhea |
| fever, cough and vomiting |
| fever and diarrhea |
| fever |
| fever and cough |
| fever and cough |
| fever and cough |
| fever and diarrhea |
| fever, cough and diarrhea |
| fever and cough |
| fever, cough and diarrhea |
| fever, cough and respiratory distress |
| fever, cough and respiratory distress |
| fever |
| fever, cough and respiratory distress |
| fever and diarrhea |
| fever |
| fever and cough |
| fever, cough and respiratory distress |
| fever and diarrhea |
| fever and cough |
| fever, cough and vomiting |
